# Lipid-Specific Labeling of Enveloped Viruses with Quantum Dots for Single-Virus Tracking

**DOI:** 10.1128/mBio.00135-20

**Published:** 2020-05-19

**Authors:** Li-Juan Zhang, Shaobo Wang, Li Xia, Cheng Lv, Hong-Wu Tang, Zhenpu Liang, Gengfu Xiao, Dai-Wen Pang

**Affiliations:** a College of Chemistry and Molecular Sciences, State Key Laboratory of Virology, The Institute for Advanced Studies, Wuhan Institute of Biotechnology, Wuhan University, Wuhan, People’s Republic of China; b Wuhan Institute of Virology, Chinese Academy of Sciences, Wuhan, People’s Republic of China; c College of Life Sciences, Henan Agricultural University, Zhengzhou, People’s Republic of China; d State Key Laboratory of Medicinal Chemical Biology, Tianjin Key Laboratory of Biosensing and Molecular Recognition, Research Center for Analytical Sciences, College of Chemistry, Nankai University, Tianjin, People’s Republic of China; Northwestern University, Feinberg School of Medicine; Columbia University/ HHMI

**Keywords:** enveloped virus, lipid-specific labeling, quantum dot, single-virus tracking

## Abstract

Quantum dots (QDs) possess optical properties of superbright fluorescence, excellent photostability, narrow emission spectra, and optional colors. Labeled with QDs, single molecules/viruses can be rapidly and continuously imaged for a long time, providing more detailed information than when labeled with other fluorophores. While they are widely used to label proteins in single-molecule-tracking studies, QDs have rarely been used to study virus infection, mainly due to a lack of accepted labeling strategies. Here, we report a general method to mildly and readily label enveloped viruses with QDs. Lipid-biotin conjugates were used to recognize and mark viral lipid membranes, and streptavidin-QD conjugates were used to light them up. Such a method allowed enveloped viruses to be labeled in 2 h with specificity and efficiency up to 99% and 98%, respectively. The intact morphology and the native infectivity of viruses were preserved. With the aid of this QD labeling method, we lit wild-type and mutant Japanese encephalitis viruses up, tracked their infection in living Vero cells, and found that H144A and Q258A substitutions in the envelope protein did not affect the virus intracellular trafficking. The lipid-specific QD labeling method described in this study provides a handy and practical tool to readily “see” the viruses and follow their infection, facilitating the widespread use of single-virus tracking and the uncovering of complex infection mechanisms.

## Abstract (precis)

Virus infection in host cells is a complex process comprising a large number of dynamic molecular events. Single-virus tracking is a versatile technique to study these events. To perform this technique, viruses must be fluorescently labeled to be visible to fluorescence microscopes. The quantum dot is a kind of fluorescent tag that has many unique optical properties. It has been widely used to label proteins in single-molecule-tracking studies but rarely used to study virus infection, mainly due to the lack of an accepted labeling method. In this study, we developed a lipid-specific method to readily, mildly, specifically, and efficiently label enveloped viruses with quantum dots by recognizing viral envelope lipids with lipid-biotin conjugates and recognizing these lipid-biotin conjugates with streptavidin-quantum dot conjugates. It is not only applicable to normal viruses, but also competent to label the key protein-mutated viruses and the inactivated highly virulent viruses, providing a powerful tool for single-virus tracking.

## INTRODUCTION

Single-particle tracking is a powerful tool to study the dynamic molecular events in living cells (1). An essential prerequisite to perform this technique is fluorescent labeling of the targets. In the past decade, various fluorescent tags such as organic dyes (2, 3), fluorescent proteins (4), metal complex of dipyridophenazine (dppz) (5), and quantum dots (QDs) (6) have been used to label the target molecules/viruses. The excellent optical properties make QDs unparalleled in single-molecule/virus tracking. Single molecules/viruses illuminated with QDs can be rapidly and continuously tracked for a long time (7), and their interactions with multiple other molecules can be monitored simultaneously (8, 9), providing more detailed information to dissect cellular events than with those labeled by other fluorophores. Thanks to these advantages, QDs have been widely used to label proteins for single-molecule-tracking studies (10–17). But due to lack of an accepted labeling method, QDs were rarely used to label viruses, which in turn limited the widespread use of single-virus tracking.

To label viruses with QDs, more than a dozen of methods have been developed, which could be roughly divided into three groups. By directly (e.g., virus-NH_2_-COOH-QD) or indirectly (e.g., virus-NH_2_-COOH-biotin-streptavidin [SA]-NH_2_-COOH-QD) attaching QDs to the amino on viral proteins, both enveloped and nonenveloped viruses can be labeled with efficiencies of 70% to 97% (group 1) (18–21). Similarly and more ingeniously, QD-labeled viruses can be obtained by genetically engineering specific viral proteins to combine them with reactive biomolecules and then with the correspondingly modified QDs (group 2) (22–24). The labeling efficiencies of this kind of method are <90%. Besides, by modifying the membranes of host cells and propagating viruses in them, viruses with reactive membranes can be harvested and then labeled with QDs (group 3) (25–28). Such methods have labeling efficiencies of 70% to 90% and labeling specificity of <90%. Although so many methods have been reported, none of them has been broadly used in practical studies due to the concerns that they may affect the bioactivity of the target proteins (group 1), they are too complicated and time consuming (group 2), or the labeling efficiency greatly varies with the cell and the virus (group 3).

The aim of this work was to provide a universal and convenient method to specifically and efficiently label enveloped viruses with QDs while preserving the native state of viral proteins. In conventional virology, lipophilic dyes such as DiO and DiD that can readily insert into lipid bilayer membranes are widely used to label viruses. Learning from the use of these long-chain lipophilic dyes, we developed a convenient method to label viruses with QDs by modifying viral lipid membranes with lipid-biotin conjugates and lighting these extraneous lipids up with SA-QD conjugates. Such a method leaves viral proteins uninvolved, and its effect on viral infectivity was negligible. It allowed enveloped Japanese encephalitis virus (JEV), porcine reproductive and respiratory syndrome virus (PRRSV), and influenza A virus (IAV) to be labeled with specificity and efficiency >95% and >93%, respectively. The whole labeling procedure comprised just five brief steps and can be performed within 2 h. With the aid of this lipid-specific QD labeling method, both wild-type (WT) and envelope (E) protein-mutated JEVs were fluorescently labeled, and their infection behaviors were thus visually analyzed.

## RESULTS AND DISCUSSION

### Labeling design.

**FIG 1:**
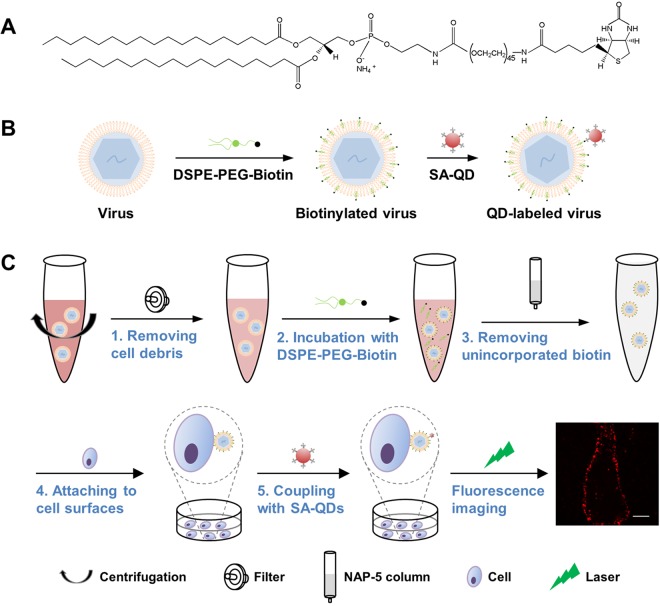
Lipid-specific QD labeling of enveloped viruses. (A) Structure of DSPE-PEG (2000)-biotin. (B) Using the rapid insertion of the lipid-biotin conjugate into lipid membranes and the specific high-affinity interaction between biotin and SA to label viruses. (C) The entire labeling procedure comprising five brief steps (1 to 5). The last panel is a fluorescence image of JEV labeled as thus on a Vero cell. Bar, 10 μm.

Labeling with high specificity and high efficiency and without affecting virus infectivity is essential to obtain high-fidelity information about virus infection, while labeling with great convenience and universal applicability is essential for a method to be widely used. To develop a QD labeling method meeting these requirements, we learned from the use of lipophilic dyes and designed a strategy to label viruses by targeting the lipid membrane. An amphipathic lipid-biotin conjugate, 1,2-distearoyl-sn-glycero-3-phosphoethanolamine (DSPE)-polyethylene glycol (PEG)-biotin (Fig. 1A), was used to recognize viral lipid membranes by the hydrophobic interaction between DSPE and lipid membranes and to mark the membranes with biotin. SA-QD conjugates were used to combine with the exogenous lipid through interaction with biotin and thus light the virus up (Fig. 1B). As seen in Fig. S1 in the supplemental material, DSPE-PEG-biotin inserted into lipid membranes as fast as DiD. After incubation with DSPE-PEG-biotin for 30 min and then with SA-QD for 10 min, cells were efficiently labeled with QDs. To apply this strategy to viruses, we optimized the labeling procedure as illustrated in Fig. 1C, clearing cell debris from the virus solution by low-speed centrifugation and syringe filtration, biotinylating viral lipid membranes by incubation with DSPE-PEG-biotin under shaking, removing unincorporated lipid-biotin molecules by gel filtration, preattaching biotinylated viruses to cell surfaces by incubation with cells at 4°C, and coupling SA-QDs to the lipid-biotin on viral membranes by incubation with the cells at 4°C. Unbound viruses and QDs were removed just by washing the cells. Such a strategy can thoroughly evade ultracentrifugation, dialysis, and ultrafiltration processes that are indispensable for removing the cell-derived reactive molecules, redundant functional reagents, unlabeled viruses, or unbound QDs in many other labeling strategies (29–34). This strategy further minimized and simplified the handling of viruses, making the QD labeling milder and more convenient. However, it should be noted that it might not apply to the viruses whose host cells cannot tolerate 4°C treatment during the labeling process.

10.1128/mBio.00135-20.2 FIG S1 Labeling cell membranes with QDs by the rapid insertion of lipid-biotin conjugates into membranes. Download FIG S1, TIF file, 2.0 MB. Copyright © 2020 Zhang et al. 2020 Zhang et al. This content is distributed under the terms of the Creative Commons Attribution 4.0 International license.

### Specifically, efficiently, and mildly labeling viruses.

**FIG 2:**
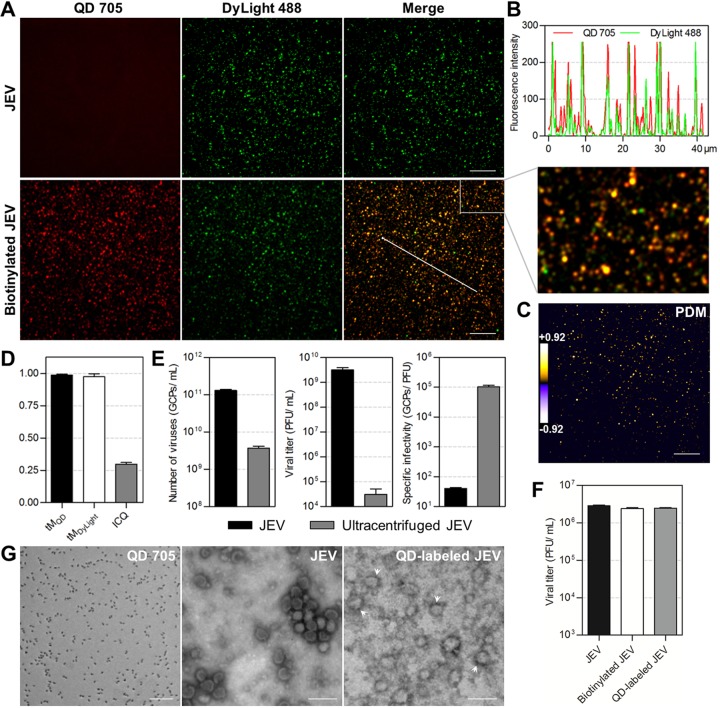
Specifically, efficiently, and mildly labeling JEV with QDs. (A) JEV and biotinylated JEV were prebound to glass slides and labeled with SA-QD 705 (red) and anti-E-DyLight 488 (green). (B) Line profile showing distributions of the signals on the line in panel A. (C) PDM image showing the colocalized (PDM > 0) and uncolocalized (PDM < 0) spots in the lower merge panel in A. Bars, 10 μm. (D) The tM_QD_, tM_DyLight_, and ICQ values calculated from 20,000 viral particles from three experiments. (E) The numbers of genome-containing particles (GCPs), titers, and specific infectivity of viruses before and after ultracentrifugation. (F) Titers of viruses before and after biotinylation and SA-QD 705 labeling (*n* = 3). (G) TEM images of SA-QD 705, JEV, and QD-labeled JEV (arrowheads). Bars, 100 nm.

JEV of approximately 50 nm in diameter was used as the model virus to experimentally evaluate the labeling strategy. Raw JEV and biotinylated JEV were prebound to glass slides and labeled with SA-QD 705 and anti-E protein-DyLight 488, respectively. As seen in Fig. 2A, there was no obvious QD signal that colocalized with DyLight-stained raw JEV, while almost all the DyLight-stained biotinylated JEV was colocalized with QDs. These data indicated that DSPE-PEG-biotin inserted into the lipid membranes of viruses, and SA-QDs efficiently bound to viruses modified with the lipid-biotin conjugate specifically through interactions with biotin. The overlapping peaks of QD and DyLight fluorescence in the line profile (Fig. 2B) and the scarcity of negative values of the product of differences from the mean (PDM) of pixel intensities in the two channels (Fig. 2C) visually showed that almost all the QD and DyLight signals were colocalized. Statistically, approximately 99% of QD signals were colocalized with the DyLight-stained viruses (ratio of the summed intensities of the QD signal in each of the pixels colocalized with DyLight signals to the total intensities of QD signals in all pixels [tM_QD_] = 0.986 ± 0.008), and approximately 98% viruses were colocalized with QDs (ratio of the summed intensities of the DyLight signal in each of the pixels colocalized with QD signals to the total intensities of DyLight signals in all pixels in thresholded images [tM_DyLight_] = 0.976 ± 0.021) (Fig. 2D). In other words, the QD labeling specificity and efficiency on glass slides were 99% and 98%, respectively. The high intensity correlation quotient (ICQ) value (0.298 ± 0.014) further confirmed this nearly complete colocalization (Fig. 2D) (35). Labeling viruses on Vero cell surfaces showed that the QD and DyLight signals still colocalized to a very high degree (94%) (see Fig. S2). The tM_QD,_ tM_DyLight,_ and ICQ values were 0.979 (± 0.018), 0.957 (± 0.030), and 0.291 (± 0.026), respectively (Fig. S2D). The specificity and efficiency of this method are superior to those of the previously reported QD labeling methods to different degrees and significantly superior to the specificity and efficiency of DiD and DiO labeling (see Fig. S3).

10.1128/mBio.00135-20.3 FIG S2 Specifically and efficiently labeling JEV with QDs on cell surfaces. Download FIG S2, TIF file, 1.8 MB. Copyright © 2020 Zhang et al. 2020 Zhang et al. This content is distributed under the terms of the Creative Commons Attribution 4.0 International license.

10.1128/mBio.00135-20.4 FIG S3 Low specificity and efficiency of DiD and DiO labeling. Download FIG S3, TIF file, 1.4 MB. Copyright © 2020 Zhang et al. 2020 Zhang et al. This content is distributed under the terms of the Creative Commons Attribution 4.0 International license.

To determine the effect of QD labeling on viruses, both the pretreatment and the labeling processes were analyzed. In our lipid-specific method, viruses were just processed with low-speed centrifugation and syringe filtration before labeling, while in many other methods, they would need further purification by ultracentrifugation (20, 36). Comparing unultracentrifuged viruses with viruses ultracentrifuged under the generally used conditions showed that high-speed centrifugation greatly reduced virus infectivity (Fig. 2E). By evading such violent pretreatment, the native infectivity of viruses was greatly preserved. During the labeling process, no cumbersome operation was performed, and no interaction involving viral proteins was used. Measuring the titer of viruses before and after QD labeling showed that the labeling process had no obvious effect on virus infectivity (Fig. 2F). As seen in the transmission electron microscope (TEM) image, QD-labeled viruses were morphologically as intact as unlabeled viruses (Fig. 2G). In aggregate, labeling viruses with QDs by the above-described lipid-specific method could preserve virus infectivity further.

### Stably and universally labeling viruses.

**FIG 3:**
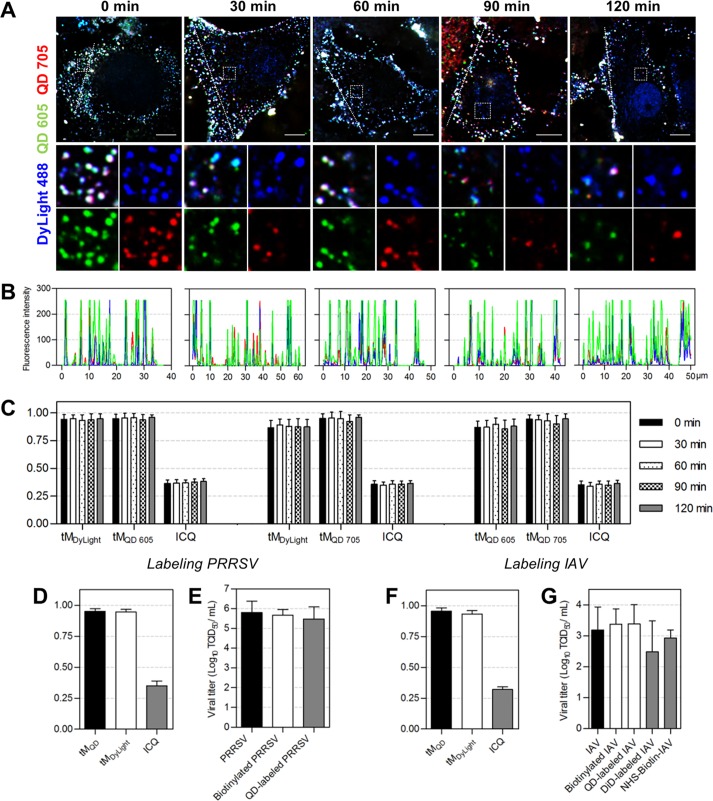
Stability and universality of the QD labeling method. (A) JEV was dually labeled with SA-QD 605 (green) and SA-QD 705 (red). Vero cells infected by the double-labeled viruses for 0, 30, 60, 90, and 120 min were fixed and stained with anti-E-DyLight 488 (blue). Bars, 10 μm. (B) Line profiles showing distributions of the fluorescence signals on the lines in panel A. (C) The tM_DyLight_/tM_QD 605_/ICQ, tM_DyLight_/tM_QD 705_/ICQ, and tM_QD 605_/tM_QD 705_/ICQ values calculated from 30 randomly selected cells. (D to G) PRRSV and IAV were labeled with QDs using the lipid-specific method. (D and F) The tM_QD_, tM_DyLight_, and ICQ values calculated from 30 cells. (E and G) Titers of viruses, biotinylated viruses, QD-labeled viruses, DiD-labeled viruses, and viruses covalently biotinylated with NHS-biotin (*n* = 3 for PRRSV and 5 for IAV).

Under the labeling conditions we used, approximately 2,836 DSPE-PEG-biotin molecules were incorporated into the lipid membranes of JEVs during biotinylation (see Fig. S4), and 2 or 3 QDs were coupled to the biotinylated virus afterwards (Fig. S5). To evaluate the stability of QDs combining with viruses, we dually labeled JEVs with QD 605 and QD 705 and allowed the viruses to infect Vero cells for different times. It was observed that the two kinds of QDs remained colocalized with the DyLight-stained viral envelope during 2 h of virus infection (Fig. 3A). Almost no QD signal was observed alone (Fig. 3B). The steady Manders’ coefficients and ICQ values of DyLight versus QD 605, DyLight versus QD 705, and QD 605 versus QD 705 suggested that the colocalization relationships among DyLight, QD 605, and QD 705 barely changed during virus infection (Fig. 3C). These results indicated that QDs coupled to viruses would not separate from the envelope and could stably point viruses out during virus infection, ensuring reliable information.

10.1128/mBio.00135-20.5 FIG S4 Quantification of DSPE-PEG-biotin on a single biotinylated JEV. Download FIG S4, TIF file, 0.7 MB. Copyright © 2020 Zhang et al. 2020 Zhang et al. This content is distributed under the terms of the Creative Commons Attribution 4.0 International license.

10.1128/mBio.00135-20.6 FIG S5 Quantification of QD 705 on a single JEV. Download FIG S5, TIF file, 0.6 MB. Copyright © 2020 Zhang et al. 2020 Zhang et al. This content is distributed under the terms of the Creative Commons Attribution 4.0 International license.

Then, we applied the above-described method to PRRSV and IAV to see how it performed when used to label other enveloped viruses. It was found that almost all the QD and DyLight used to label PRRSV colocalized with each other, with tM_QD_, tM_DyLight_, and ICQ values of 0.950 (± 0.022), 0.946 (± 0.022), and 0.352 (± 0.037), respectively (Fig. 3D and S6). The infectious titers of biotinylated PRRSV and QD-labeled PRRSV were nearly the same as that of the raw PRRSV (Fig. 3E), suggesting that QD labeling did not affect PRRSV infection. When used to label IAV, the method still showed high specificity and efficiency (tM_QD_ = 0.955 ± 0.028, tM_DyLight_ = 0.933 ± 0.027, and ICQ = 0.320 ± 0.022) (Fig. 3F and S7). Comparing DiD labeling and the QD labeling based on covalent interactions with amino acids on viral surfaces (37, 38), the lipid-specific QD labeling method was superior for preserving virus infectivity (Fig. 3G). These results demonstrated that the method described in Fig. 1 is universally applicable for the specific, efficient, and mild labeling of enveloped viruses.

10.1128/mBio.00135-20.7 FIG S6 Specifically and efficiently labeling PRRSV with QDs. Download FIG S6, TIF file, 0.9 MB. Copyright © 2020 Zhang et al. 2020 Zhang et al. This content is distributed under the terms of the Creative Commons Attribution 4.0 International license.

10.1128/mBio.00135-20.8 FIG S7 Specifically and efficiently labeling IAV with QDs. Download FIG S7, TIF file, 1.5 MB. Copyright © 2020 Zhang et al. 2020 Zhang et al. This content is distributed under the terms of the Creative Commons Attribution 4.0 International license.

### Imaging the infection of WT and mutant JEVs.

**FIG 4:**
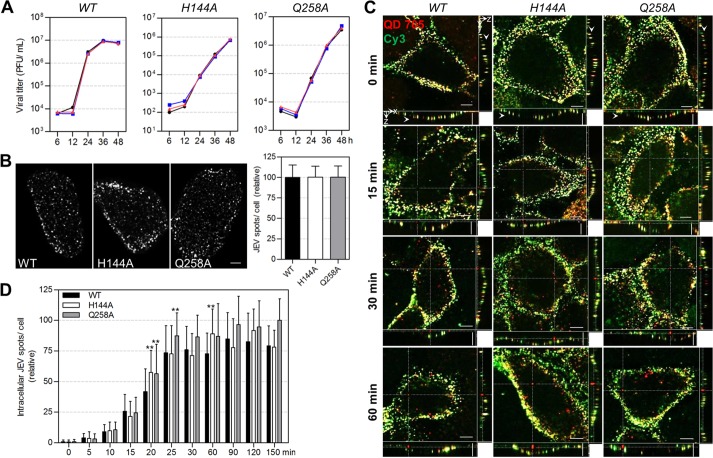
The entry activity of WT, H144A, and Q258A JEVs. (A) One-step growth curves of WT, H144A, and Q258A JEVs (*n* = 2). The black, blue, and red lines are the curves of raw, biotinylated, and QD-labeled viruses, respectively. (B) WT and mutant JEVs were attached to Vero cell surfaces and labeled with QD 705. Cells were imaged in three dimensions (3D) and analyzed with Fiji software. The left panels are the z-projection images of cells attached with WT, H144A, and Q258A JEVs. The histogram is the QD-labeled WT/H144A/Q258A JEV spots per cells (*n* = 100). (C and D) WT and mutant viruses were labeled with QD 705 and allowed to infect cells for 0, 5, 10, 15, 20, 25, 30, 60, 90, 120, and 150 min. Then, the viruses that remained on cell surfaces were stained with Cy3. After fixation, the cells were imaged in 3D and analyzed with Velocity software. (C) Cells infected for the indicated times. Horizontal and vertical scale bars, 10 μm. (D) Numbers of viruses internalized in cells after infection for different times (*n* = 30).

JEV E protein on the envelope plays essential roles in virus infection. In our previous work, site mutations were introduced to the E protein, and several amino acids were proved to be important for the virus membrane fusion (39). But their roles in the virus transport within cells remain unresolved, since it is difficult to study the dynamic trafficking of viruses by traditional methods. Here, we labeled the WT, H144A mutant, and Q258A mutant JEVs with QDs to visually analyze the effect of the two substitutions on virus infection. The nearly overlapping one-step growth curves of the viruses before and after QD labeling showed that QD labeling had no evident effect on the infectivity of WT, H144A, and Q258A JEVs (Fig. 4A). To analyze the virus entry activity, the same amounts of WT and mutant viruses were bound to cell surfaces (Fig. 4B) and allowed to infect cells at 37°C for different times. Then, the cells were immediately transferred to 4°C and incubated with SA-Cy3 to additionally stain the QD-labeled viruses remaining on cell surfaces, making them distinguishable from the QD-labeled viruses internalized in the cells (Fig. 4C). By counting the viruses solely labeled with QDs, the amount of viruses inside cells was determined. It was found that after synchronization at 4°C, most WT viruses entered cells in the first 25 min, and the number of viruses inside cells plateaued in the next 2 h (Fig. 4D), consistent with the viruses’ internalization kinetics into B104 cells (40). H144A and Q258A viruses followed similar entry kinetics to that of the WT virus. Except for individual time points, the amounts of mutant viruses internalized into cells at most time points were similar to those of the WT virus, indicating that replacing the H144 and Q258 amino acids in the E protein with alanines did not affect JEV uptake into Vero cells.

**FIG 5:**
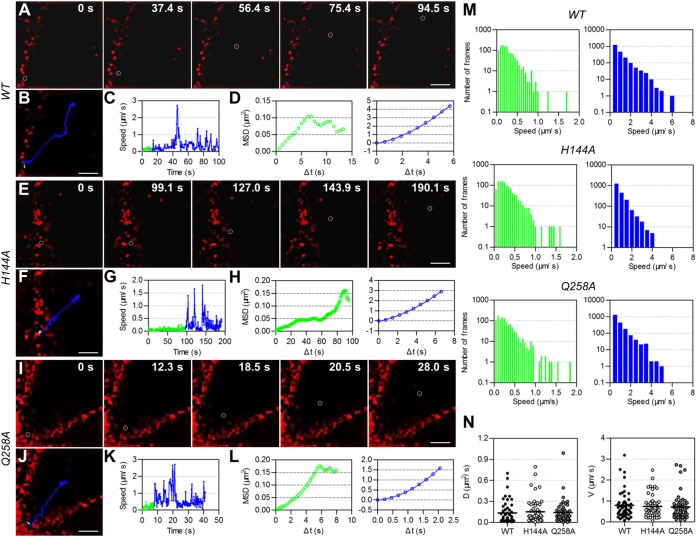
Intracellular transport behaviors of WT, H144A, and Q258A JEVs. QD-labeled WT/H144A/Q258A JEV virions were allowed to infect living Vero cells at 37°C and imaged in real time by a spinning-disk confocal microscope. (A, E, and I) Snapshots of QD-labeled viruses (red) infecting cells. (B, F, and J) Trajectories of the circled viruses in panels A, E, and I. (C, G, and K) Speed versus time plots of the viruses. (D, H, and L) MSD versus Δ*t* plots of the viruses (green and blue symbols). The green symbols cannot be fitted. The blue lines are the fits to MSD = 4*D*Δ*t* + (*V*Δ*t*)^2^ with *D *= 0.081/0.053/0.039 μm^2^/s and *V *= 0.29/0.19/0.55 μm/s. *D* and *V* are the diffusion coefficient and mean velocity, respectively. (M) Statistics of the instantaneous speeds of viruses. (N) Statistics of the *D* and *V* of WT/H144A/Q258A JEV moving actively.

Then, we visually analyzed the transport behaviors of WT and mutant JEVs in the cytoplasm after their entry via endocytosis by tracking individual QD-labeled virions. The dynamic transport process of single WT viruses from the cell periphery toward the interior region was observed (Fig. 5A). It was divided into two stages, viruses moving slowly and irregularly in the cell periphery (green lines in Fig. 5B to D) and those moving rapidly and actively toward the interior of cells (blue lines in Fig. 5B to D), according to the viruses’ speeds, relationships between mean square displacement (MSD) and Δ*t* (time interval), and location in cells. As indicated by drug inhibition, the infection of JEV and its rapid active motion in Vero cells were dependent on microtubules and dynein while independent of microfilaments (Fig. S8). Dynein is the molecular motor protein responsible for powering cargo moving along microtubules toward the cell nucleus (41). Therefore, virus motion in the second stage was the process by which dynein drove JEV-carrying endosomes to move along microtubules toward the interior region, consistent with our previous findings that dynein directionally drove IAV-carrying endosomes along microtubules during virus infection (42, 43). On the other hand, among the known motions, the anomalous or confined motion on cell membranes and the slow active motion on microfilaments were reported occurring before the rapid active motion on microtubules (42, 44), both of which differed from the slow irregular motion found here. Considering that the intermediate filament network has been found to physically hinder the transport of organelles (45), we speculated that the dense actin network could also hinder the transport of vesicles. The slow irregular motion of JEV was the process by which the virus-carrying endosomes diffused across the dense actin-rich region near the plasma membrane. Tracking the movement of single H144A and Q258A JEV virions showed that the two types of mutant viruses moved toward the cell interior in a similar two-stage pattern (Fig. 5E to L). Statistically analyzing virus speeds in the two stages revealed that H144A and Q258A JEVs moved with speeds of <1.0 μm/s in the first stage and with speeds up to several microns per second in the second stage, just as the motion of WT viruses (green and blue histograms in Fig. 5M). The diffusion coefficients and mean velocities of H144A and Q258A viruses in the second stage had no significant differences with those of the WT viruses (Fig. 5N). These results indicated that these two substitutions in the E protein did not affect the intracellular transport behaviors of JEV.

10.1128/mBio.00135-20.9 FIG S8 JEV transport via a microfilament-independent and microtubule/dynein-dependent pathway. Download FIG S8, TIF file, 1.8 MB. Copyright © 2020 Zhang et al. 2020 Zhang et al. This content is distributed under the terms of the Creative Commons Attribution 4.0 International license.

**FIG 6:**
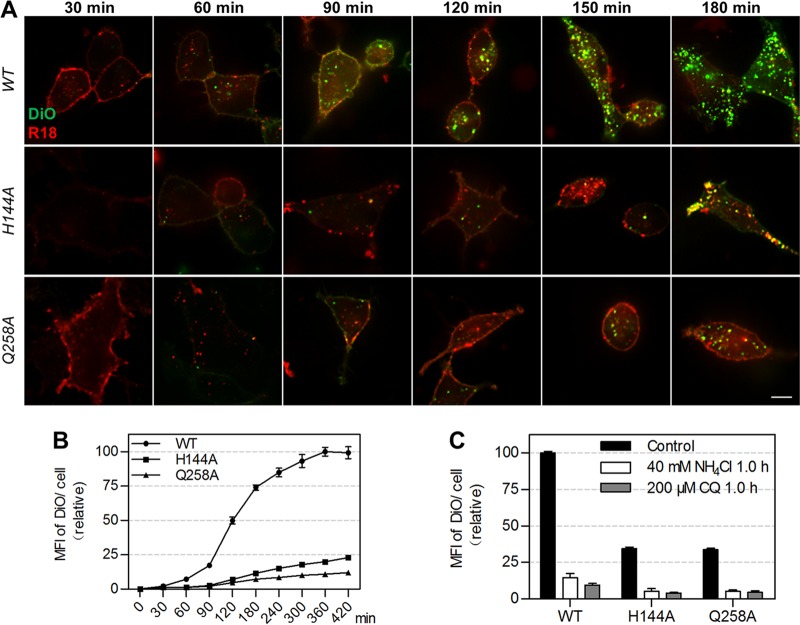
The fusion activity of WT, H144A, and Q258A JEVs. (A) DiO/R18 double-labeled viruses were allowed to infect Vero cells for different times. Bar, 10 μm. (B) Mean fluorescence intensities (MFIs) of DiO in cells infected by the double-labeled viruses for different times measured by flow cytometry (*n* = 3). (C) MFIs of DiO in cells treated with drugs and infected by JEV for 1 h. NH_4_Cl and chloroquine (CQ) were used to block virus-endosome fusion (*n* = 3).

Sequentially, we analyzed the fusion activity of the mutant JEVs. Since it was difficult to follow the membrane fusion process of viruses using the above-described QD labeling method, a previously reported dual-wavelength imaging method was used in this part (46). The viruses were dually labeled with lipophilic DiO and R18 at concentrations allowing them to be solely illuminated by R18 before virus membrane fusion and simultaneously illuminated by R18 and DiO after membrane fusion (46). Thus, the fusion of virus membranes with acidic endosome membranes could be determined by measuring the fluorescence intensity of DiO (47). As seen in Fig. 6A, the amount of WT viruses fused with endosomes was greater than that of H144A and Q258A viruses. The fluorescence intensity of DiO in the cells infected by WT viruses increased rapidly in the second and third hours and plateaued gradually in the following 4 h, while the DiO fluorescence in cells infected by mutant viruses increased very slowly (Fig. 6B). The amounts of H144A and Q258A viruses fused with endosomes were just 23% and 12% of WT viruses after infection for 7 h. In the presence of low-pH inhibitors, the fluorescence intensity of DiO in cells infected by WT and mutant viruses reduced at the same degree (Fig. 6C). These results indicated that H144A and Q258A substitutions reduced the membrane fusion activity of JEV. Taken together, H144 and Q258 are dispensable for the uptake and intracellular transport of JEV but essential for its membrane fusion with endosomes.

### Conclusion.

We developed a lipid-specific method to mildly, readily, specifically, and efficiently label enveloped viruses with QDs. The unique optical properties of QDs, the high specificity and efficiency, and the comparative convenience make them superior to DiD and DiO labeling. The advantages in convenience and universality make this lipid-specific method prevail over other QD labeling methods. More importantly, since the target molecules are lipids, this method is capable of labeling key protein-mutated viruses, which is significant for in-depth study of virus infection mechanisms. Because this labeling method does not involve virus propagation, it can also be used to study inactivated highly virulent viruses such as HIV and Ebola virus. The labeling technique described in this study provides a powerful tool to visually investigate the dynamic infection of enveloped viruses.

## MATERIALS AND METHODS

### Cells.

Vero, Madin-Darby canine kidney (MDCK), baby hamster kidney (BHK-21), and MARC-145 cells were maintained in Dulbecco’s modified Eagle’s medium (DMEM; Gibco) supplemented with 10% fetal bovine serum (FBS; South American origin, PAN Biotech) under 5% CO_2_ at 37°C.

### Viruses.

JEV SA-14-14-2 and WT JEV AT31 were propagated in BHK-21 cells. H144A and Q258A mutant JEV virions were packaged using cDNA clones of JEV AT31 as described previously (39). PRRSV (HN07-1 strain) was propagated in MARC-145 cells. Collected JEV- and PRRSV-containing cell culture supernatants were centrifuged at 1,500 rpm and 4°C for 10 min and filtered with 0.2-μm-pore-size filters (Millipore) to remove cell debris. To evaluate the effect of ultracentrifugation on virus infectivity, part of the JEV SA-14-14-2 sample was further purified by ultracentrifugation (48, 49). In brief, viruses were concentrated by centrifugation at 100,000 × *g* and 4°C in a Ty45 Ti rotor (Beckman) for 2 h, purified by gradient centrifugation on 10% to 35% potassium tartrate-glycerol (30%) at 125,000 × *g* in a SW32 Ti rotor for 2 h, and then desalted at 180,000 × *g* in the Ty45 Ti rotor for 1 h. IAV [A/chicken/Hubei/01-MA01/1999(H9N2) strain] was propagated in pathogen-free chicken eggs and purified by sucrose gradient ultracentrifugation as described previously (50). All the harvested viruses were subpackaged and stored at −80°C until use.

### Virus labeling.

Viruses were incubated with 30 μM DSPE-PEG (2000)-biotin (Avanti) at room temperature for 1 h. Unincorporated biotin and aggregated viruses were removed by NAP-5 gel filtration columns (GE Healthcare) and 0.2-μm-pore-size filters, respectively. Then, biotinylated viruses and 2 nM SA-QD 705 (Wuhan Jiayuan Quantum Dots Co., Ltd.) were successively incubated with cells at 4°C for 30 and 10 min, respectively, allowing viruses to prebind to cell surfaces and QDs to bind to viruses. Unbound viruses and QDs were removed by washing cells with ice-cold phosphate-buffered saline (PBS). To track virus infection, the cells were immediately warmed to 37°C and imaged on a spinning-disk confocal microscope equipped with a cell culture system.

To stain the QD-labeled viruses on cell surfaces with Cy3, cells infected by QD-labeled viruses were immediately shifted to 4°C and incubated with 2 nM SA-Cy3 (Thermo) for 10 min, allowing Cy3 to bind to the QD-labeled biotinylated viruses through the interaction between SA and biotin. After fixation with ice-cold paraformaldehyde and washing with PBS, cells with QD-labeled viruses in the interior and Cy3-QD-double-labeled viruses on the surface were imaged on the confocal microscope.

Labeling of viruses with DiD/DiO was performed by incubating viruses with 5 μM DiD/DiO (Beyotime Biotechnology) while shaking and in the dark at room temperature for 1 h. Labeling of viruses with both DiO and R18 was performed by incubating viruses with 0.2 μM DiO and 0.4 μM R18 (Millipore) under the same conditions. Unbound dyes and aggregates were removed by gel filtration and syringe filtration.

### Immunofluorescence assay.

Anti-Japanese encephalitis E (mouse monoclonal; Millipore), influenza A H9N2 HA (mouse monoclonal; Sino Biological Inc.), and PRRSV nucleocapsid protein (rabbit monoclonal; VMRD) antibodies were used to localize JEV, IAV, and PRRSV, respectively. DyLight 488/649-conjugated secondary antibodies (Abbkine) were used to label the primary antibodies, illuminating the viruses.

### Virus infectivity.

The infectious infectivity of JEV and the number of genome-containing particles (GCPs) were measured by plaque assay on BHK-21 cells and quantitative PCR (qPCR) as described previously (39). To compare the specific infectivity of JEV and ultracentrifuged JEV, ultracentrifuged viruses were resuspended to the original volume after ultracentrifugation. Then, the GCP and PFU in the samples with or without ultracentrifugation were measured. The specific infectivity was determined by dividing GCPs by the number of PFU. PRRSV infectivity was measured by TCID_50_ on Vero cells. IAV infectivity was measured by 50% tissue culture infective dose (TCID_50_) assay on MDCK cells and hemagglutination assay on red blood cells (51). *N*-Hydroxysuccinimide (NHS)-biotin-IAV was obtained as described previously (50). Briefly, 100 μl of IAV was incubated with 0.1 mg Sulfo-NHS-long-chain (LC)-biotin (Thermo) at room temperature for 2 h. Unbound biotin and aggregates were removed by filtration.

### TEM imaging.

Twenty microliters of 10 nM SA-QD 705, JEV, and biotinylated JEV incubated with 0.1 nM SA-QD 705 were dropped on carbon-coated copper grids. After 0.5 h (for SA-QD 705) or 15 h (for JEV and QD-labeled JEV) at 4°C, the grids were drained by using filter papers and washed with ultrapure water. After being stained with sodium phosphotungstate for 3 min (for JEV) or 30 s (for QD-labeled JEV), the grids were air dried and imaged on a Hitachi-7000FA transmission electron microscope.

### Fluorescence imaging.

Fluorescence images were captured by a spinning-disk confocal microscope (Andor Revolution XD). Hoechst 33342, DyLight 488/DiO, R18, and DyLight 649/DiD/CellMask deep red plasma membrane stain were imaged using 405-, 488-, 561-, and 640-nm lasers (DPSS Lasers Inc.) and 447/60-, 525/50-, 605/20-, and 685/40-nm emission filters (Chroma), respectively. QD 605 and QD 705 were imaged using the 488-nm laser and 605/20- and 685/40-nm emission filters.

### Image analysis.

Colocalization events were statistically evaluated by thresholded Manders’ coefficient and intensity correlation analysis (ICA) using ImageJ (35, 52). Regions of interest (ROIs) were used to perform the analysis. Manders’ coefficients vary from 0 (nonoverlapping images) to 1 (100% colocalized images) and are termed tM_QD_ and tM_DyLight_ here according to the image names. tM_QD_ is the ratio of the summed intensities of the QD signal in each of the pixels colocalized with DyLight signals to the total intensities of QD signals in all pixels in thresholded images, and tM_DyLight_ is defined conversely. ICA is based on the assumption that the summed difference of pixel intensities from the mean in a single channel is zero, namely, ∑_n pixels_ (*I*_QD, i_ − *I*_QD, mean_) = 0 and ∑_n pixels_ (*I*_DyLight, i_ − *I*_DyLight, mean_) = 0. PDM is the product (*I*_QD, i_ − *I*_QD, mean_)(*I*_DyLight, i_ − *I*_DyLight, mean_). Intensity correlation plots show the intensity as a function of PDM. ICQ is the ratio of the summed positive PDM from two channels to the total PDM subtracted by 0.5. It varies from −0.5 (mutual exclusion) to +0.5 (complete colocalization) and indicates a strong covariance in the range from 0.1 to 0.5 (53). Line profiles of signals were acquired with Image-Pro Plus.

Trajectories of viruses were reconstructed by linking points in each frame using the nearest-neighbor association and the motion history of individual particles with Image-Pro Plus (54, 55). MSD representing the average squared distance of all steps within a trajectory for Δ*t* (Δ*t* = *τ*, 2*τ*, 3*τ*, and so on, *τ* = acquisition time interval between frames) was calculated using MATLAB (56). Modes of motion were analyzed by fitting MSD and Δ*t* to functions MSD = 4*D*Δ*t* (normal or Brownian diffusion), MSD = 4*D*Δ*t* + (*V*Δ*t*)^2^ (active or directed diffusion), and MSD = 4*D*Δ*t^α^* (anomalous diffusion) (57).

### Statistical analysis.

Data are represented as means ± standard deviations (SD). Student's *t* tests were performed for all statistical analyses with the original un-normalized data. Statistical significance was determined by two-tailed *P* values.

10.1128/mBio.00135-20.1 TEXT S1 Descriptions of methods and figures. Download Text S1, DOCX file, 0.1 MB. Copyright © 2020 Zhang et al. 2020 Zhang et al. This content is distributed under the terms of the Creative Commons Attribution 4.0 International license.
