# Supplementary material for: Lipid-Specific Labeling of Enveloped Viruses with Quantum Dots for Single-Virus Tracking
Source: mBio. 2020 May 19;11(3):e00135-20. doi: 10.1128/mBio.00135-20 (PMC7240151; doi:10.1128/mBio.00135-20)
Supplement: TEXT S1 [file mBio.00135-20-s0001.docx]

Supporting information for

Lipid-specific labeling of enveloped viruses with quantum dots for single-virus tracking

Li-Juan Zhang,^1¶^ Shaobo Wang,^2¶^ Li Xia,^1^ Cheng Lv,^1^ Hong-Wu Tang,^1^ Zhenpu Liang,^3^ Gengfu Xiao,^2*^ Dai-Wen Pang^1,4*^

^1^College of Chemistry and Molecular Sciences, State Key Laboratory of Virology, The Institute for Advanced Studies, and Wuhan Institute of Biotechnology, Wuhan University, Wuhan, People's Republic of China

^2^Wuhan Institute of Virology, Chinese Academy of Sciences, Wuhan, People's Republic of China

^3^College of Life Sciences, Henan Agricultural University, Zhengzhou, People's Republic of China

^4^State Key Laboratory of Medicinal Chemical Biology, Tianjin Key Laboratory of Biosensing and Molecular Recognition, Research Center for Analytical Sciences, and College of Chemistry, Nankai University, Tianjin, People's Republic of China

^*^Corresponding Author

Email: dwpang@whu.edu.cn (D.-W. P.); xiaogf@wh.iov.cn (G. X.)

^¶^Li-Juan Zhang and Shaobo Wang contributed equally to this work. Author order was determined through consultation.

**Supplemental methods**

**Cell membrane labeling.** Vero cells cultured in glass bottom dishes were incubated with 30 μM DSPE-PEG-Biotin at room temperature for 0, 0.5, 1.0, 2.0, 3.0, and 4.0 h. After removal of unincorporated biotin, the cells were incubated with 2 nM SA-QD 605 for 10 min. Then cells were fixed and labeled with CellMask Deep Red plasma membrane stain and Hoechst 33342 (Invitrogen). After washing, the cells were imaged on the confocal microscope. For the flow cytometric analysis, cells cultured in 24-well plates were biotinylated and labeled with QD 705 in the same way. After trypsinization and fixation, the cells were suspended in 500 μL of PBS and analyzed by flow cytometry (BD LSRFortessa). Parallelly, cells were incubated with DiD for different time and analyzed by microscopy and flow cytometry.

**Quantification of biotin on JEV.** DSPE-PEG-Biotin incorporated into virus membranes was quantified as described (1, 2). In brief, 2 mL of 2 nM SA-FITC (Sigma-Aldrich) was taken in a stirred cuvette to which biotin was added with increments of 0.5 pmol at 10 min-intervals until the fluorescence intensity of FITC became steady. Fluorescence spectra of the SA-FITC solution were recorded by a fluorescence spectrometer (Horiba Jobin Yvon), from which the relationship between SA-FITC fluorescence intensity and biotin consumption was determined. In the same way 5 uL of JEV and biotinylated JEV were added to SA-FITC solution and the corresponding fluorescence spectra were recorded. According to the relationship between SA-FITC fluorescence and biotin, the amount of DSPE-PEG-Biotin on biotinylated JEV was determined. By determining the number of JEV virions by qPCR (3), the lipid-biotin conjugate on each biotinylated JEV was quantified.

**Quantification of QDs on JEV.** SA-QD 705 was dispersed on glass bottom dishes, processed with 4% paraformaldehyde, and imaged with a 50 ms exposure. QD-labeled JEV was bound to cell surfaces, and fixed and imaged under the same conditions as SA-QD 705. Gray levels of the QD spots and the QD-labeled virus spots were analyzed with Velocity software. The number of QDs on each JEV was roughly determined as the ratio of 'the gray level of QD-labeled viruses' to 'the gray level of single QDs'. The gray levels of QD-labeled viruses and single QDs were subtracted by those of the background.

**Supplemental figure legends**

**FIG S1** Labeling cell membranes with QDs by the rapid insertion of lipid-biotin conjugates into membranes. (A) Cells incubated with 5 μM DiD (red) for 0, 30, 60, 120, 180, and 240 min were imaged by a confocal microscope. (B) Cells were incubated with 30 μM DSPE-PEG-Biotin for 0, 30, 60, 120, 180, and 240 min and then with SA-modified QDs for 10 min (red). CellMask Deep Red Plasma Membrane Stain (green) and Hoechst 33342 (blue) were used to stain the plasma membrane and the nucleus. Scale bars, 10 μm. (C, D) Mean fluorescence intensity (MFI) of the DiD/QD-labeled cells and the labeling efficiency measured by flow cytometry (n = 3).

**FIG S2** Specifically and efficiently labeling JEV with QDs on cell surfaces. (A) JEV and biotinylated JEV were pre-attached to cell surfaces and labeled with SA-QDs (red) at 4°C. After fixation, viruses on cell surfaces were further labeled with anti-E-DyLight 488 (green). Scale bars, 10 μm. (B) Line profile showing distributions of the QD and DyLight signals on the line in A. (C) PDM image showing the colocalized and uncolocalized spots in the lower merge panel in A. (D) The tM_QD_, tM_DyLight_, and ICQ values calculated from 30 randomly selected cells.

**FIG S3** Low specificity and efficiency of DiD and DiO labeling. (A, D) DiD/DiO-labeled JEV (red) were attached to Vero cell surfaces at 4°C and further labeled with anti-E-DyLight 488/649 (green) after fixation. Images were acquired by the same confocal microscope setup. Scale bars, 10 μm. (B, E) Signals in the overlapped images in A and D were randomly connected with lines. The line profiles show distributions of DiD/DiO and DyLight signals on the lines. (C, F) The tM_DiD/DiO_, tM_DyLight_, and ICQ values calculated from 40 randomly selected cells.

**FIG S4** Quantification of DSPE-PEG-Biotin on single biotinylated JEV. (A) Schematic representation of the method used to quantify biotin with SA-FITC conjugates. (B) Fluorescence spectra of SA-FITC solution titrated with biotin. Lines from the bottom to the top show the biotin consumption of 0, 0.5, 1.0, 1.5, 2.0, 2.5, 3.0, 3.5, 4.0, 4.5, and 5.0 pmol. (C) The fluorescence intensity of SA-FITC at 515 nm. (D) The dependence between fluorescence intensity of SA-FITC and the biotin consumption (symbols). The red line is the fit to y = 209677x + 238115. The fluorescence intensity of SA-FITC has good linear relation with biotin consumption in the range from 0.5 to 2.5 pmol. (E) Fluorescence spectra of SA-FITC solution added with 0.5 pmol biotin, 5 μL of JEV, and 5 μL of biotinylated JEV. (F) The amount of DSPE-PEG-Biotin on single JEV virions (n = 3).

**FIG S5** Quantification of QD 705 on single JEV. (A) Fluorescence spectra of QD 705 and QD-labeled JEV. This data indicated that combining with viruses didn't change QD fluorescence. (B) Statistic gray levels of QD 705 subtracted by that of the noise. The red line is the fit to Gaussian function and the mean is 449.8, indicating that the gray level of most QD particles is about 450. (C) The trace of a QD with gray level of about 450 (left) and a zoom of it (right), showing that this QD particle has obvious blinking behaviors and is a single QD. (D) About 95% QDs with gray levels of about 450 are single QD (n = 113). Results from B−D suggest that the gray level of single QD is around 450. (E) Statistic gray lavels of QD-labeled JEV subtracted by that of brackground, showing that the gray level of most virions is around 900 and 1350. That means most JEV virions combined with 2 or 3 QDs.

**FIG S6** Specifically and efficiently labeling PRRSV with QDs. (A) PRRSV was biotinylated with DSPE-PEG-Biotin, attached to Vero cell surfaces, and then labeled with SA-QD 705 (red). After fixation, PRRSV was further labeled with anti-PRRSV-DyLight 488 (green). (B) Intensity correlation plots (ICPs) of the QD and DyLight signals in A and their scatter plot. The C-shaped curves of dots in ICPs and the centred dots in the scatter plot show that QD and DyLight signals are almost completely colocalized. (C) PDM image of the double-labeled viruses showing the colocalized and uncolocalized spots. (D) Line profile showing distributions of the QD and DyLight signals on the line in the overlapped image of A.

**FIG S7** Specifically and efficiently labeling IAV with QDs. (A) IAV was biotinylated with DSPE-PEG-Biotin, attached to MDCK cell surfaces, and labeled with SA-QD 705 (red). After fixation, IAV was further labeled with anti-HA-DyLight 488 (green). (B) ICPs of QD and Dylight signals and the scatter plot. (C) PDM image showing the colocalized and uncolocalized spots in the overlapped image of A. (D) Line profile showing distributions of the signals on the line in A.

**FIG S8** JEV transport *via* a microfilament-independent and microtubule/dynein-dependent pathway. (A) QD-labeled JEV was allowed to infect Vero cells treated with 0.2% DMSO, 20 μM cytochalasin D (CytoD), 60 μM nocozadole (Noc), and 100 μM ciliobrevin D (CilioD). CytoD, Noc, and CilioD were used to block microfilaments, microtubules, and dynein, respectively. After 0.5 h of virus uptake, viruses remained on cell surfaces were stained with SA-Cy3 at 4°C to be distingushed from the internalized viruses. After fixation, the cells were imaged in 3D and analyzed with Velocity. Horizontal scale bars, 10 μm. Vertical scale bars, 5 μm. (B) The amount of viruses internalized in cells treated with drugs (n = 50). (C) Virus infection in cells treated with drugs were tracked. The white lines are trajectories of viruses. (D) The speed *vs.* time plots of the viruses tracked in C. (E) The MSD *vs.* *Δt* plots (black symbols). The upward lines in the first two graphs are the fits to MSD = 4*DΔt* + (*VΔt*)^2^ with D = 0.031/0.014 μm^2^/s and V = 0.088/0.10 μm/s. The downward line in the third graph is the fit to MSD = 4*DΔt^α^* with D = 0.0012 μm^2^/s and α = 0.77.

**References**

1. Hu J, Wen CY, Zhang ZL, Xie M, Xie HY, Pang DW. 2014. Recognition kinetics of biomolecules at the surface of different-sized spheres. Biophys J 107:165-173.
2. Lv C, Lin Y, Liu AA, Hong ZY, Wen L, Zhang Z, Zhang ZL, Wang H, Pang DW. 2016. Labeling viral envelope lipids with quantum dots by harnessing the biotinylated lipid-self-inserted cellular membrane. Biomaterials 106:69-77.
3. Liu HB, Liu Y, Wang SB, Zhang YJ, Zu XY, Zhou Z, Zhang B, Xiao GF. 2015. Structure-based mutational analysis of several sites in the E protein: Implications for understanding the entry mechanism of Japanese encephalitis virus. J Virol 89:5668-5686.
